# Isolated Peptide from Spider Venom Modulates Dendritic Cells In Vitro: A Possible Application in Oncoimmunotherapy for Glioblastoma

**DOI:** 10.3390/cells12071023

**Published:** 2023-03-27

**Authors:** Felipe Cezar de Mato, Natália Barreto, Gabriel Cordeiro, Jaqueline Munhoz, Amanda Pires Bonfanti, Thomaz A. A. da Rocha-e-Silva, Rafael Sutti, Priscilla B. M. Cruz, Livia R. Sanches, André Luis Bombeiro, Ghanbar Mahmoodi Chalbatani, Liana Verinaud, Catarina Rapôso

**Affiliations:** 1 Faculdade de Ciências Farmacêuticas, Universidade Estadual de Campinas (UNICAMP), Campinas 13083-871, SP, Brazil; 2 Departamento de Biologia Estrutural e Funcional, Instituto de Biologia, UNICAMP, Campinas 13083-862, SP, Brazil; 3 Department of Agricultural, Food and Nutritional Sciences (AFNS), University of Alberta, Edmonton, AB T6G 2R3, Canada; 4 Department of Physiological Sciences, Faculdade Israelita de Ciências da Saúde Albert Einstein, São Paulo 05653-120, SP, Brazil; 5 Valer Laboratórios Eireli, São Paulo 13347-633, SP, Brazil; 6 Department of Immunology, Mayo Clinic, Scottsdale, AZ 85259, USA

**Keywords:** cell therapy, immunoadjuvant, dendritic cells vaccine, *Phoneutria nigriventer*, PnV, glioblastoma

## Abstract

Dendritic cells (DCs) vaccine is a potential tool for oncoimmunotherapy. However, it is known that this therapeutic strategy has failed in solid tumors, making the development of immunoadjuvants highly relevant. Recently, we demonstrated that *Phoneutria nigriventer* spider venom (PnV) components are cytotoxic to glioblastoma (GB) and activate macrophages for an antitumor profile. However, the effects of these molecules on the adaptive immune response have not yet been evaluated. This work aimed to test PnV and its purified fractions in DCs in vitro. For this purpose, bone marrow precursors were collected from male C57BL6 mice, differentiated into DCs and treated with venom or PnV-isolated fractions (F1—molecules < 3 kDa, F2—3 to 10 kDa and F3—>10 kDa), with or without costimulation with human GB lysate. The results showed that mainly F1 was able to activate DCs, increasing the activation-dependent surface marker (CD86) and cytokine release (IL-1β, TNF-α), in addition to inducing a typical morphology of mature DCs. From the F1 purification, a molecule named LW9 was the most effective, and mass spectrometry showed it to be a peptide. The present findings suggest that this molecule could be an immunoadjuvant with possible application in DC vaccines for the treatment of GB.

## 1. Introduction

Dendritic cells (DCs) are antigen-presenting cells (APCs) that have distinct characteristics according to their degree of maturation [1]. When activated (mature), DCs express high levels of proteins related to antigen presentation, for example, major class II histocompatibility complex (MHCII) and surface costimulatory molecules such as CD80 and CD86, allowing for an effective mechanism of antigen presentation to T cells [2]. In addition, mature DCs secrete anti- or pro-inflammatory cytokines, which control T cell differentiation, effectively regulating the adaptive immune response [3,4].

DCs play a significant role in the maintenance of the innate immune system in infectious conditions of invading pathogens, as well as against tumors, highlighting their relevant role as a natural sentinel. Such functions are largely mediated by classical cytokines produced by DCs, which are commonly released in response to multiple signals and stimuli. Some pro-inflammatory cytokines, such as TNF-α and IL-1β, have an extensive and direct involvement in the regulation of innate immunity, while other cytokines produced by DCs, such as IL-10 and IL-12, have specific opposite functions, acting in the polarization of the response of T cells, as well as helping to control the inflammatory process. In addition, it is noted that some cytokines, such as IFNs, in certain circumstances, play a dual role, promoting a protective and rapid immune response through their direct effects on the replication of the pathogen or tumor cells, or through regulatory actions of immune functions in certain defense cells, such as DCs [5]. Therefore, it is clear that the general balance of available cytokines in the microenvironment and the direct interaction between these molecules with DCs are considered fundamental aspects that influence and drive the immune reaction profile [5].

Thus, due to their ability to direct the response, DCs have been identified as a potential therapeutic tool for immunotherapies, a strategy that has gained prominence in the field of oncology [6,7,8,9]. The main oncoimmune approaches include monoclonal antibodies against checkpoint molecules (such as cytotoxic T lymphocyte–associated antigen-4—CTLA-4; programmed cell death-1—PD-1 and PD-1 ligand—PD-L1), cytokines, adoptive transfer T cells engineered to express chimeric antigen receptors (CARs), vaccines using DCs, and potentially others [6,7]. However, despite numerous clinical studies evaluating therapeutic vaccination in cancer during the last two decades, this method has not been used successfully in many types of tumors, especially so-called “cold tumors” (high reduction of infiltrating T-cells in the interior or in the tumor margins), such as glioblastomas (GBs) and other solid neoplasms [6,8,9].

Among the approaches to improve immunotherapies, adjuvants act by inducing the maturation of DCs, increasing the effectiveness of vaccines [3,5]. Studies have shown that molecules from spider or scorpion venoms have immunomodulatory effects [10,11,12]. It was demonstrated by our research group that the venom of *Phoneutria nigriventer* (PnV; Ctenidae, Araneomorphae), a spider found in South America, decreased cell viability and proliferation, impaired the cell cycle and induced apoptosis of human GB lineages and PnV purified components impaired cell migration and adhesion through RhoA-ROCK and Na^+^/K^+^-ATPase [13,14]. In addition, the venom reduced or eradicated GB development in a preclinical trial using a murine model [15]. Bonfanti et al. (2020) also showed that the systemic administration of PnV increased circulating monocytes and NK (Natural Killer) cells and augmented the number of intra and peritumoral macrophages. These results lead us to investigate whether PnV components activate immune cells. A recent study demonstrated that, in fact, the venom induced a phagocytic and active macrophage profile [16]; however, there is no information so far concerning the effects of PnV on the adaptive immune system. In this sense, the present study aims to evaluate the effect of PnV and its purified components as adjuvants in the activation of DCs, focusing on the treatment of GB.

## 2. Materials and Methods

### 2.1. Animals and Ethical Considerations

All experiments were conducted in accordance with the Ethical Principles on Animal Research, adopted by the Brazilian College of Animal Experimentation (Colégio Brasileiro de Experimentação Animal, COBEA), and all procedures with animals were approved by the Ethics Committee on the Use of Animals (CEUA; #5172-1/2019) from the State University of Campinas (UNICAMP). In this work, C57BL/6 adult female mice (eight weeks old) were used. All mice were kept in regular filter-top cages, with free access to sterile food and water, and maintained in the animal facility of the Department of Genetics, Evolution, Microbiology and Immunology at UNICAMP.

### 2.2. Reagents and Phoneutria nigriventer Venom (PnV)

All chemicals were purchased from Sigma-Aldrich (St. Louis, MO, USA) unless otherwise noted. Two samples of lyophilized PnV were obtained by electrical stimulation of numerous adult spiders (male and female) (National System Management of Genetic Patrimony and Associated Traditional Knowledge—Sisgen—registry #A551346). The quality and reproducibility of the venom were evaluated by high-pressure liquid chromatography (HPLC). Lyophilized venom, fractions and subfraction were stored at −80 °C and dissolved in phosphate buffered saline (PBS) immediately before use.

### 2.3. Venom Purification

The crude venom fractionation was performed using a 10 kDa nominal cut-off Amicon^®^ filter (Millipore, Billerica, MA, USA #UFC801008) by centrifugation. This process consisted of separating the components of the venom (10 mg dissolved in distilled water) by molecular weight using molecular filters, generating three main fractions named: F1 (low weight, less than 3 kDa), F2 (intermediate weight, between 3 and 10 kDa) and F3 (high weight, more than 10 kDa). 

After the tests described below, F1 and F2 together were referred to as the LW (low-weight; <10 kDa) and F3 was referred as the HW (high-weight; >10 kDa). The LW fraction was subsequently chromatographed by reversed-phase HPLC on a C_18_ column (ACE, Aberdeen, Scotland) using aqueous 0.1% trifluoroacetic acid as the mobile phase and 90% acetonitrile in the mobile phase as the eluent with a gradient run from 0% to 100% over 100 min. This step separated venom components in ten subfractions referred to as LW1 to LW10. The more active peak obtained in this second step (LW9) was fractionated by cation exchange HPLC on a Luna SCX column (Phenomenex, Torrance, CA, USA) equilibrated with 0.05 M potassium phosphate, pH 2.5, and eluted with a linear gradient of 0–1 M KCl as the mobile phase for 40 min. The resulting peak was desalted and sent to mass spectrometry analysis. In both chromatographic steps, the elution profiles were monitored at 214 nm and 280 nm. Mass spectrometry was performed in a Xevo G2-XS QTof (Waters, Framingham, MA, USA) in negative mode with ramp collision energy from 20 to 30 V.

### 2.4. Obtaining DCs

Mice were euthanized by overdose of the association of dissociative anesthetic (ketamine, 300 mg/Kg) and alpha 2 adrenoreceptor agonist (xylazine, 30 mg/Kg) administered intraperitoneally. Bone marrow precursors were collected from the tibia and femur, and the cell suspension was adjusted to a concentration of 2 × 10^5^ cells/mL and seeded in a 96-well plate. Cells were maintained for seven days (culture medium was changed every other day) at 37 °C and 5% CO_2_ in Iscove’s Modified Dulbecco’s Medium (IMDM), containing 10% fetal bovine serum (FBS), 50 μg/mL gentamicin (Gibco, ThermoFisher Scientific Inc., Waltham, MA, USA) and supplemented with 10 ng/mL Granulocyte-Macrophage Colony-Stimulating Factor (GM-CSF; Biolegend, San Diego, CA, USA; #505406) [17]. This protocol differentiates precursor cells in conventional/myeloid DCs. Supplementary Figure S1 shows the gate strategy and percentages of DCs obtained from precursor cells.

### 2.5. Culture of Human (NG97) and Murine (GL261) GB Cells and Obtaining Lysate

The human GB (NG97) cells were donated by a patient at Hospital das Clínicas/UNICAMP and the cell line was established and characterized in a sequence of published studies [18,19,20,21]. Cells were seeded at a density of 1 × 10^4^ per cm^2^ in a 25 cm^2^ culture bottle and grown in IMDM supplemented with 10% FBS, 100 IU/mL penicillin and streptomycin (pH 7.4) (Gibco/ThermoFisher Scientific Inc., Waltham, MA, USA #10378016). The GL261 cell lineage was donated by the Department of Cancer Biology at Thomas Jefferson University. Cells were cultivated in RPMI 1640 medium (Gibco/ThermoFisher Scientific Inc. # 11875-101) with 10% fetal bovine serum (FBS), 0.5 mg/mL L-Glutamine, Penicillin, Streptomycin (Gibco/ThermoFisher Scientific Inc.) and 0.05 mM Mercaptoethanol (Gibco/ThermoFisher Scientific Inc. #21985023). Cells were maintained at 37 °C and 5% CO_2_ until complete confluence. To obtain the lysate, after confluence, the cells were released from the bottles with cell scraper and transferred to a Falcon tube, being then centrifuged at 1500 revolutions per minute (RPM) for 5 min at 4 °C. After centrifugation, the supernatant was discarded and the pellet was re-suspended in 1 mL IMDM supplemented with 10% FBS for cell counting. The ratio of one DC for every five NG97 or GL261 cells (1:5) was used. After counting the cells, the necessary volume was transferred to an eppendorf tube, frozen in liquid nitrogen for 10 min and thawed in a bath at 37 °C (the process was repeated three times). Cells were centrifuged for 15 min at 12,000 RPM and 4 °C, the supernatant (cell lysate) was collected and adjusted to the appropriate volume and stored in a biofreeze at −80 °C [22].

### 2.6. Treatment of DCs In Vitro

After seven days of differentiation, DCs received the following treatments: control group (unstimulated), *E. coli* O26:B6 LPS 1 µg/mL (used as a positive control for DCs activation), crude PnV 14 µg/mL, PnV fractions (F1, F2 and F3, 1 µg/mL), or purified subfractions from F1 + F2 (LW1-10, 1 µg/mL). All groups were performed without or with concomitant stimulation with human (NG97) or murine (GL261) GB cells lysate. Currently, there are some ways to obtain tumor-specific activation of DCs, for example, using molecules isolated or synthesized from tumor cells, or using the total content of lysed tumor cells. Several clinical studies to date demonstrated that tumor-specific activation of DCs using total tumor cell lysate is an effective methodology, capable of inducing a potent and extensive immune response against tumors and providing a lower risk of immune escape by tumor cells [23,24,25,26,27,28]. For the cell viability test with thiazolyl tetrazolium bromide (MTT) only, cells were treated with PnV at 1.4, 14, 140 or 280 µg/mL and fractions (F1, F2 and F3) at 1 µg/mL (all without GB cells lysate). DCs were incubated with the treatments at 37 °C and 5% CO_2_ for 24 h. All groups were performed in triplicate, in three independent experiments for each method described below.

### 2.7. Cell Viability Test (MTT)

MTT was used to determine whether PnV and its fractions have cytotoxic effects on DCs. For this, cells were treated with PnV at different concentrations (1.4, 14, 140 or 280 µg/mL) or F1, F2 and F3 (1 µg/mL) in a 96-well plate and incubated for 24 h at 37 °C and 5% CO_2_. Then, treatments were removed and cells were added with 100 μL of IMDM supplemented with 10% FBS, 100 IU/mL of penicillin and streptomycin (pH 7.4) and 10 μL of MTT, and incubated at 37 °C for 4 h, according to the manufacturer’s protocol (Sigma-Aldrich, EUA—#MKBP6775V). After, the medium with MTT was removed and 100 µL of acidified isopropanol was added to each well to solubilize the blue MTT crystals. Plates were read at 570 nm on a Multiskan™ GO microplate spectrophotometer (Thermo Fisher Scientific, Inc., Waltham, MA, USA).

### 2.8. Detection of Surface Markers by Flow Cytometry

After treating the DCs with PnV (14 µg/mL) or fractions (1 µg/mL) for 24 h (with or without GB cells lysate), aliquots containing 2 × 10^5^ cells were resuspended and incubated for 30 min at 4 °C in 100 μL of PBS containing 2% of rat serum. Cells were then labeled with 20 μL of a cocktail containing the following antibodies against surface molecules, according to the manufacturer’s protocol (1:20; eBioscience, San Diego, CA, USA): MHCII (Clone M5/114.15.2; PE Cy7; #25-5321-80), CD11c (Clone N418; APC; #117310), CD80 (Clone 16-10A1; FITC; #104706) and CD86 (Clone GL1; PE; #12-0862-82). After, the cells were fixed with 4% paraformaldehyde for 20 min at room temperature in a dark environment. Analyses were performed in a flow cytometer (100.000 events/sample) (BD FACSVerse), and data evaluated by FlowJo VX software (Tristar Inc., Santa Rosa Beach, FL, USA).

## 3. Measurement of Cytokine Production by Enzyme-Linked Immunosorbent Assay (ELISA)

Quantification of TNF-α (tumor necrosis factor-alpha), IL (Interleukin)-1β and IL-10 in the culture medium (DC supernatant) was performed using Biolegend immunoassay kits according to the manufacturer’s instructions. Monoclonal antibodies against TNF-α, IL-1β and IL-10 (#B244585, #B236998 and #B246139, respectively) were previously incubated in the microplate. Standards, controls and samples were added to the wells, and cytokines were bound to the immobilized antibody. After washing, a substrate solution was added. The enzymatic reaction produces a blue product that turns yellow when the stopping solution is added. The measured color intensity is proportional to the amount of cytokines bound. The values were read by the standard curve in a Multiskan™ GO microplate spectrophotometer (Thermo Fisher Scientific, Inc., Waltham, MA, USA).

### 3.1. Morphological Analysis

Considering that morphology is indicative of the DC profile, in order to analyze the immunomodulatory effect of PnV and fractions, cells were examined by microscopy. After differentiation and treatments for 24 h, morphological records were performed using Cytation 5—Cell Imaging Multi-Mode Reader (BioTek, Winooski, VT, USA) and Eclipse Ts2 Diascopic- Inverted Microscope (Nikon Instruments Inc., Melville, NY, USA). Cells were evaluated by two observers, considering the shape of the body and the presence of processes (indicative of an activated profile).

### 3.2. Statistical Analysis

Values were analyzed using the GraphPad Prism software package (San Diego, CA, USA; v. 5.0). The level of significance was obtained by one-way analysis of variance (ANOVA) and the Kruskal–Wallis test. Data are presented as mean ± standard error (SEM). An unpaired Student’s *t*-test was used to compare each treatment with the control. A *p*-value < 0.05 indicates statistical significance.

## 4. Results

### 4.1. Reproducibility of the Venom, Purifications and Toxicity of Fractions

**Figure 1:**
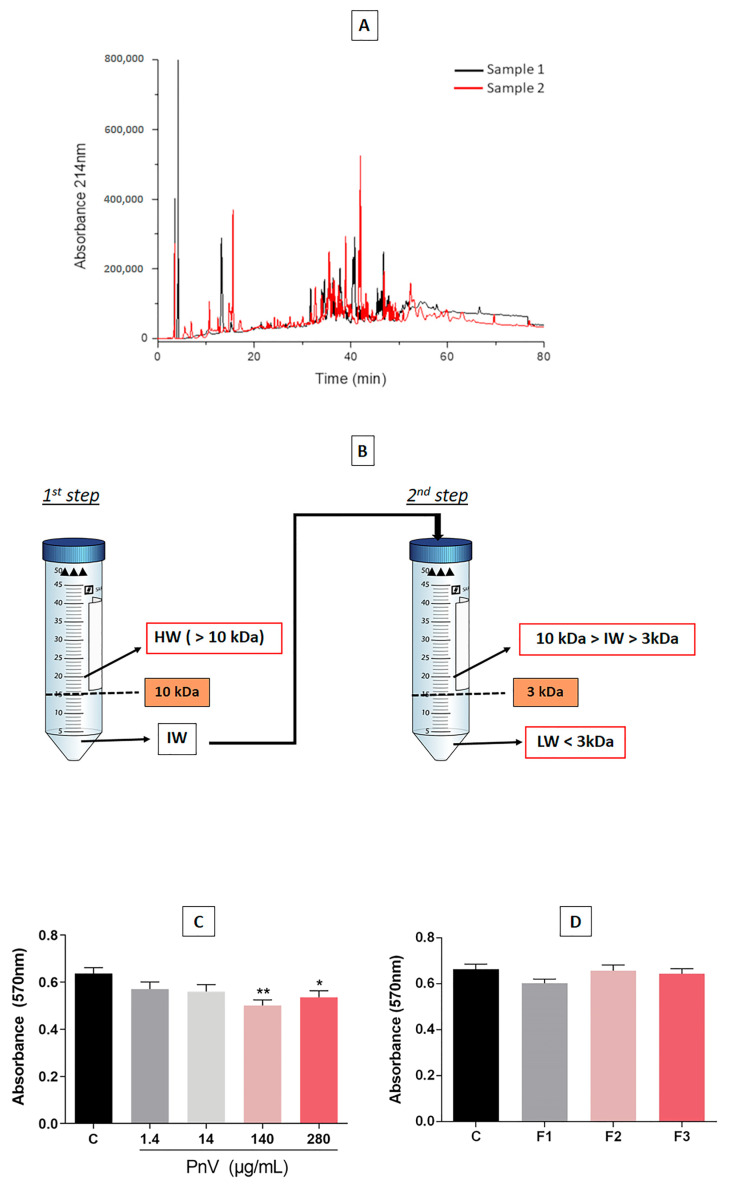
PnV profile obtained by high-pressure liquid chromatography (HPLC) and cell viability assay. The HPLC showed that there were no relevant differences between the two pooled venom samples used in this work (**A**). Stages of the first fractionation of PnV by molecular mass: HW = high weight (F3); IW = intermediate weight (F2); LW = low weight (F1) (**B**). Cell viability (MTT) analysis by the effect of PnV at different concentrations (1.4, 14, 140 and 280 µg/mL) and fractions (F1, F2 and F3; 1 µg/mL) in DCs in vitro after twenty-four hours incubation (**C**,**D**). Data were analyzed by one-way ANOVA and Kruskal–Wallis test. Unpaired Student’s *t*-test was used to compare each treatment with the control. * *p* < 0.05 and ** *p* < 0.01 compared to the untreated control. Data are presented as mean ± standard error (SEM). Results of three independent experiments.

As shown in Figure 1A, the chromatogram confirms the quality and purity of the PnV, demonstrating that there was no appreciable difference between the two extracted venom samples. Figure 1B illustrates the procedure performed to separate components of the crude venom by molecular weight, obtaining three different fractions (F1, F2 and F3).

Treatment with high concentrations of PnV (140 and 280 μg/mL) significantly decreased the viability of DCs, showing cytotoxic activity after 24 h of exposure. However, no statistically significant change in cell viability was observed when used at lower concentrations (1.4 and 14 μg/mL) (Figure 1C). No significant changes were observed when DCs were treated with purified PnV fractions (F1, F2 and F3; 1 µg/mL) for 24 h (Figure 1D).

**Figure 2:**
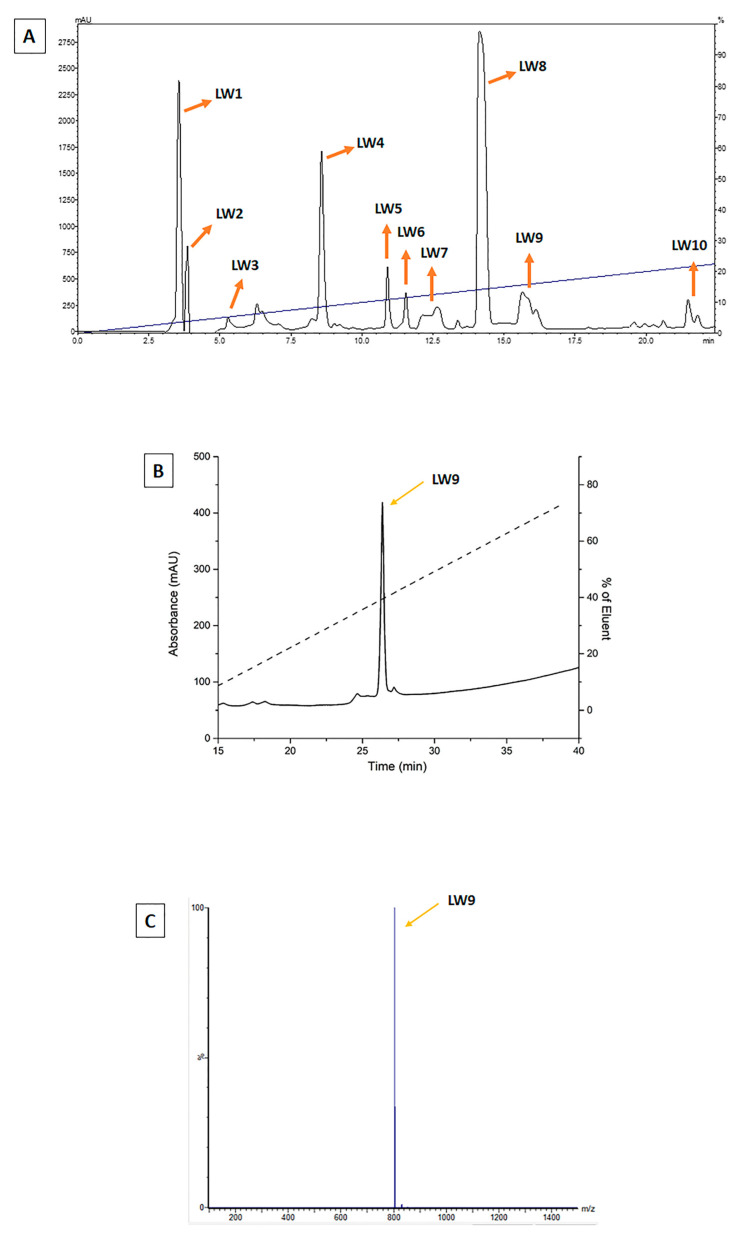
Purification profile of molecules isolated from PnV fractions by high-pressure liquid chromatography (HPLC). Panel (**A**) shows the HPLC purification of PnV fractions F1 (low weight) and F2 (intermediate weight), yielding ten different subfractions (LW1–LW10). (**B**,**C**) show the ion exchange chromatographic profile and the mass spectroscopy, respectively, of LW9, which revealed that the fraction have a satisfactory degree of purity.

As described below, F1 showed the most promising effects. Therefore, this fraction was purified by HPLC (F1 was pooled with F2, as F2 had very few components and was difficult to purify alone), generating 10 subfractions, which were named Low Weight—LW1 to LW10 (Figure 2A). These PnV-components were tested for adjuvant effects on the DCs and LW9 was considered the most promising, as shown below. Then, LW9 was subjected to ion chromatography (Figure 2B) and mass spectrometry (Figure 2C), showing that this subfraction is composed by a single peptide, whose structure will be further characterized. The Supplementary Figure S2 shows the chromatographic profile of the two samples of pooled F1 and F2 (panels A and B) and F3 (panels C and D), demonstrating that the two lots of samples used in this work have similar compositions. The F3-components were not tested as F1 showed the best effects in the first screening.

### 4.2. F1 Increased Surface Molecules of DCs When Used Concomitantly with the NG97 Cells Lysate

**Figure 3:**
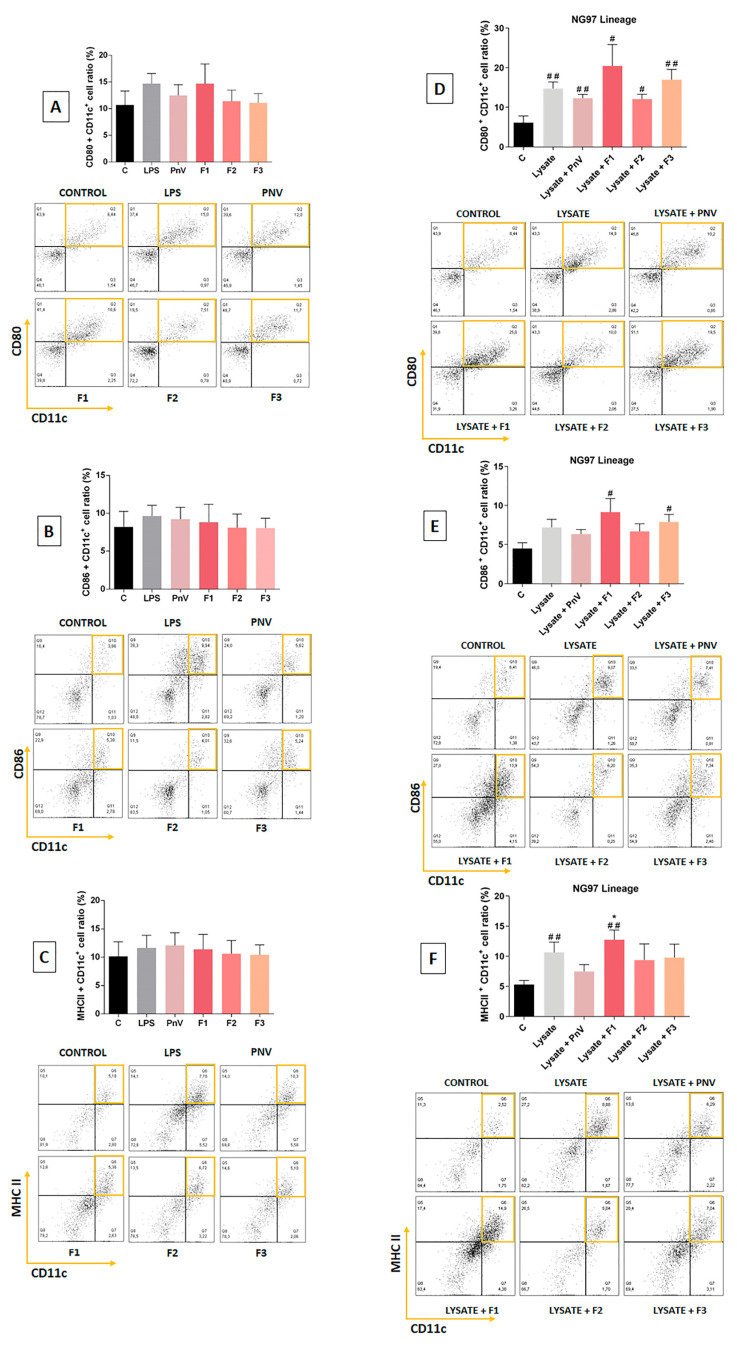
Flow cytometry of DC surface markers. Percentage of DCs expressing the surface markers CD80, CD11c, CD86 and MHC II in vitro. Cells were stimulated with LPS, PnV (14 µg/mL) or fractions (1.0 µg/mL) for 24 h (**A**–**C**). Graphs (**D**–**F**) show the markers in DCs stimulated with glioblastoma cells (NG97) lysate and simultaneously treated with PnV (14 µg/mL) or fractions (1.0 µg/mL) for 24 h. The panels behind the graphs show representative double positive gates of each group. Data were analyzed by one-way ANOVA and Kruskal–Wallis test, presented as mean ± standard error (SEM). Unpaired Student’s *t*-test was used to compare each treatment with the control. # *p* < 0.05, ## *p* < 0.01 compared to the untreated control; * *p* < 0.05 compared to the lysate plus PnV group. Results of three independent experiments.

The DCs treated with PnV and its fractions without GB cell lysate (Figure 3A–C) showed no alteration in surface markers (MHCII, CD80, CD86 and CD11c) compared to the control (untreated cells). LPS also induced no significant change. When the DCs were exposed only to human (NG97) GB cell lysate (Figure 3D–F), all markers were increased compared to untreated control, being statistically significant in CD80 and MHCII. 

No treatment with venom or fractions had significant differences compared to lysate alone. Treatment with lysate concurrently with PnV did not differ from lysate alone for all surface markers, indicating that the observed increase compared to untreated control was induced by lysate and not by PnV. However, F1 plus lysate increased CD86 significantly compared to the untreated control, whereas lysate alone had no statistically significant difference. Treatment with F2 plus lysate makes the difference of CD80 and MHCII compared to the untreated control less statistically significant than lysate alone versus the untreated control. Treatment with F3 plus lysate induced a significant increase in CD86 compared to untreated control, whereas lysate alone induced no significant difference. The panels behind the graphs show representative double positive gates for each group.

### 4.3. F1 and F2 Increased Cytokines Release When Used with Simultaneous NG97 Cells Lysate

**Figure 4:**
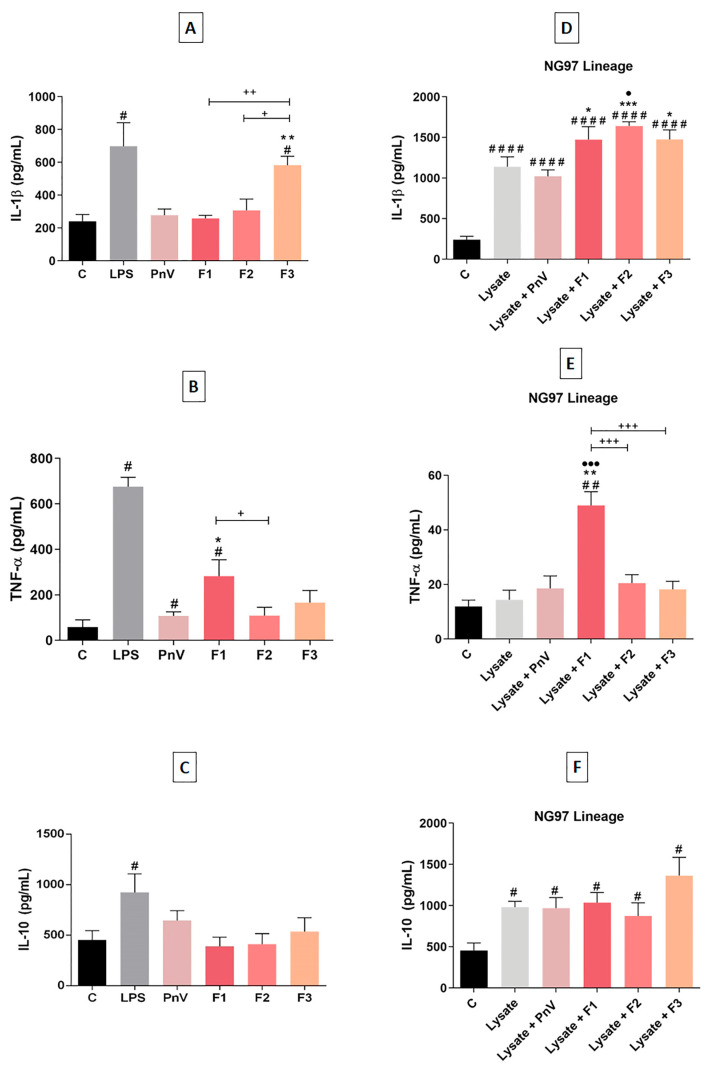
ELISA quantification of cytokines released by DCs in culture. Analysis of pro-inflammatory and anti-inflammatory cytokines (IL-1β, TNF-α and IL-10) in the supernatant of DCs after stimulation with PnV (14 µg/mL) and fractions (1.0 µg/mL), with or without simultaneous glioblastoma cells (NG97) lysate, for 24 h. (**A**–**C**) Without concomitant lysate stimulation. (**D**–**F**) With concomitant lysate stimulation. Data were analyzed by one-way ANOVA and Kruskal–Wallis test, presented as mean ± standard error (SEM). Unpaired Student’s *t*-test was used to compare each treatment with the control. # *p* < 0.05 compared to the untreated control; # *p* < 0.05, ## *p* < 0.01, #### *p* < 0.0001 compared to the untreated control * *p* < 0.05, ** *p* < 0.01, *** *p* < 0.001 compared to the lysate plus crude PnV group; ● *p* < 0.05, ●●● *p* < 0.001 compared to the lysate group; + *p* < 0.05, ++ *p* < 0.01, +++ *p* < 0.001 compared between fractions. Results of three independent experiments.

When cells were exposed to treatments without NG97 cells lysate (Figure 4A–C), LPS induced a significant increase in all cytokines (IL1β, TNF-α and IL-10) compared to the untreated control; LPS was used as a positive control of DCs activation and results showed that cells were able to be activated by a stimulus. F3 significantly increased IL-1β compared to control, PnV, F1 and F2. PnV and F1 significantly increased TNF-α compared to untreated cells. In addition, F1 significantly increased TNF-α compared to the treatment with PnV and F2. Neither PnV or fractions altered IL-10 release.

Treatment with NG97 cell lysate alone (Figure 4D–F) induced a statistically significant increase in IL-1β and IL-10, but did not alter TNF-α when compared to control group. Exposure to PnV plus lysate added no effect to all cytokines examined; IL-1β and IL-10 (but not TNF-α) still increased compared to untreated DCs and this effect can be attributed to the lysate and not to PnV. All fractions enhanced the effect of the NG97 cells lysate, significantly increasing IL-1β release compared to untreated DCs and to lysate plus PnV. Furthermore, only F2 plus lysate was significantly increased compared to lysate alone. F1 plus lysate significantly increased TNF-α compared to all other groups. Regarding IL-10 release, no treatment added effect to the lysate exposure, with all groups being significantly greater than control, but not compared to lysate alone.

All fractions, without and with simultaneous exposure to NG97 cells lysate, induced a morphological profile of activated DCs.

**Figure 5:**
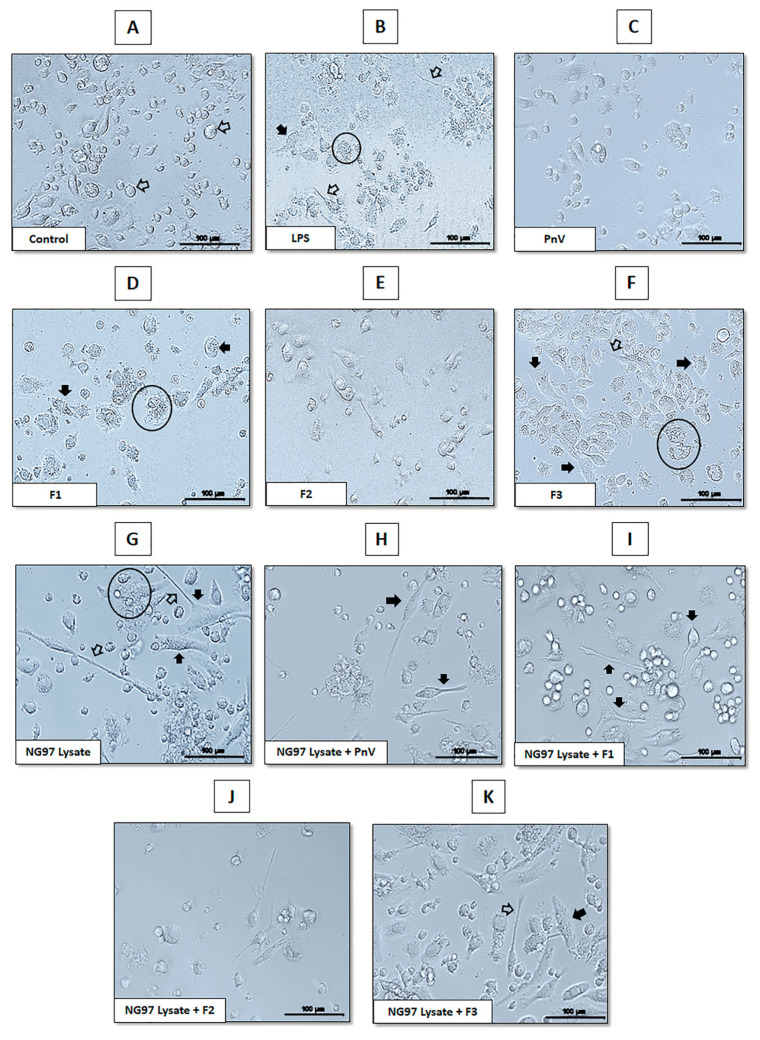
Analysis of morphological changes in DCs. Representative panels of morphological analysis of DCs treated for 24 h with PnV (14 µg/mL) or its isolated molecules (1 µg/mL), with or without simultaneous stimulation with glioblastoma cells (NG97) lysate. Signs in panels indicate morphological changes compared to the untreated control (panel (**A**)). (**B**–**K**) represent all treatments, as indicated in each panel. Filled arrows: irregular bodies; unfilled arrows: processes; circle: cells with granular cytoplasm. Morphological analyses were made using the Cytation-5 (BioTeK). Bars = 100 µm.

Representative panels of morphological analyses are shown in Figure 5. This tool has been used in association with other parameters to demonstrate the activation of DCs [28,29,30]. The classic morphology of differentiated DCs that received no stimulus (immature DCs) is rounded cell bodies with very few processes (unfilled arrows in panel A). When treated with LPS, cells showed a typical mature morphology, with enlarged and more irregular bodies (filled arrows in panel B), processes (unfilled arrows at panel B) and a granular cytoplasm (see circled cell at panel B). The treatment with PnV did not induce relevant changes comparing to untreated control, being very similar to this group (panel C). The exposure to F2 (panel E) induced discrete changes in DCs, with fewer bigger bodies and few more processes than untreated cells (panel A). On the other hand, exposure of DCs to F1 and F3 (panels D and F, respectively) induced similar characteristics of LPS treatment (panel B), with enlarged and irregular bodies (filled arrows), processes (unfilled arrows) and cytoplasm with granules (circled cells). 

When DCs were exposed only to NG97 cells lysate (panel G), morphology was typically activated. Simultaneous treatment with lysate and PnV or lysate and F2 (panels H and J, respectively) induced a slightly less activated profile compared to lysate alone (smaller cells with fewer processes). On the other hand, F1 and F3 (panels I and K) induced a very activated morphology; however, the profile, interestingly, was little different than lysate alone, with apparently more cells showing mature characteristics, without the granular aspect of cytoplasm. See in all panels filled arrows showing cellular bodies, unfilled arrows pointing to processes and circles showing granular cells. 

All fractions (mainly F1 and F3), with simultaneous exposure to murine (GL261) GB cells lysate, increased cytokine production and induced an activated morphological profile of the DCs.

**Figure 6:**
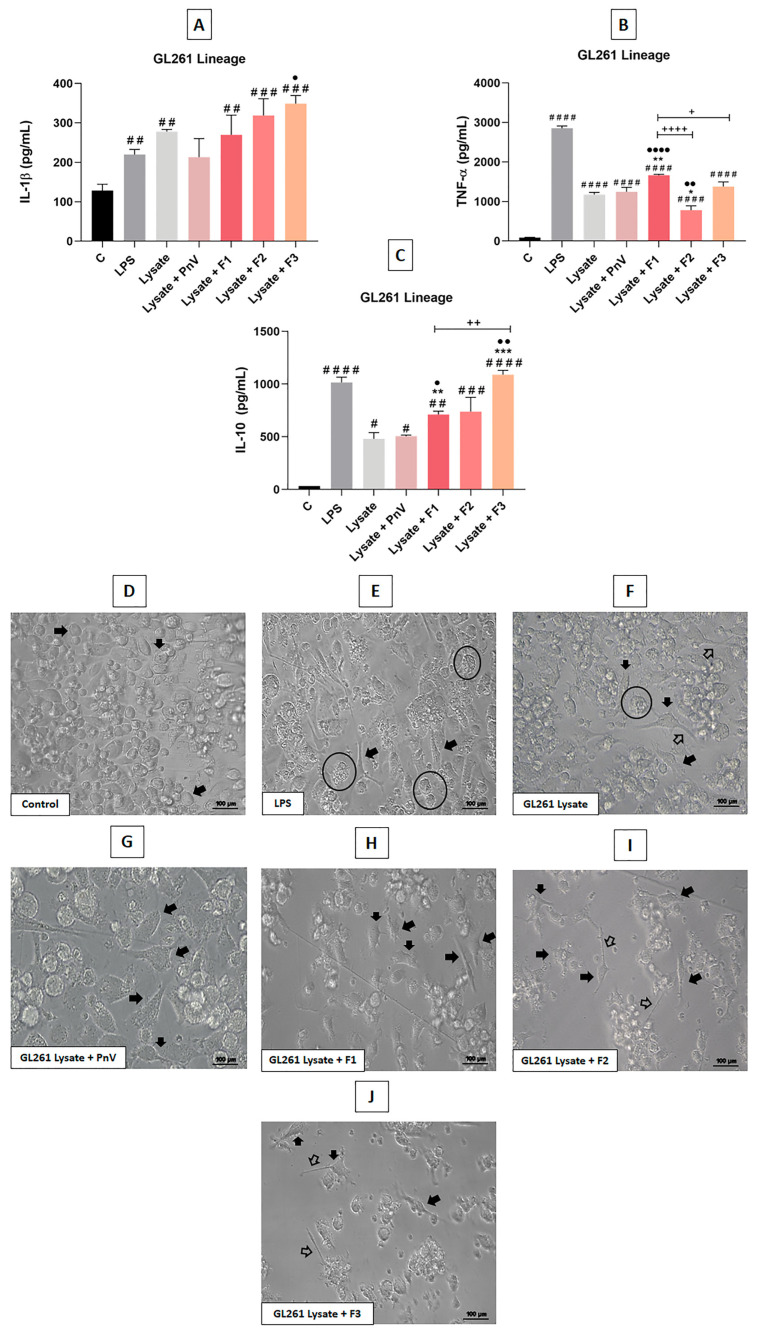
Cytokines release and morphology of DCs stimulated with glioblastoma cells (GL261) lysate. Graphs (**A**–**C**) show production of pro-(IL-1β, TNF-α) and anti-inflammatory (IL-10) cytokines by DCs after stimulation with PnV (14 µg/mL) or its fractions (1 µg/mL), with or without simultaneous murine glioblastoma cells (GL261) lysate (GL261), for 24 h (detection by ELISA). Panels (**D**–**J**) show morphological changes observed in DCs after treatments. Signs in panels indicate morphological changes compared to the untreated control (panel (**D**)). Filled arrows: irregular bodies; unfilled arrows: processes; circle: cells with granular cytoplasm. Eclipse Ts2 Diascopic (Nikon Inc.); Bars = 100 µm. Data were analyzed by one-way ANOVA and Kruskal–Wallis test, presented as mean ± standard error (SEM). Unpaired Student’s *t*-test was used to compare each treatment with the control. # *p* < 0.05, ## *p* < 0.01, ### *p* < 0.001, #### *p* < 0.0001 compared to the untreated control; * *p* < 0.05, ** *p* < 0.01, *** *p* < 0.001 compared to the murine GB lysate plus PnV group; ● *p* < 0.05, ●● *p* < 0.01, ●●●● *p* < 0.0001 compared to the lysate group; + *p* < 0.05, ++ *p* < 0.01, ++++ *p* < 0.0001 compared between fractions. Results of two independent experiments.

When the DCs were exposed to murine GB cell lysate (Figure 6A–C), all cytokines (IL1β, TNF-α and IL-10) were increased compared to the untreated control. Positive control with LPS also induced a significant increase in all cytokines. All treatments, except PnV plus lysate, significantly increased IL-1β compared to the untreated control. However, only F3 plus lysate induced a significant increase in this cytokine compared to lysate alone (Figure 6A). Regarding TNF-α, all treatments induced a significant increase compared to the untreated control, but only F1 plus lysate significantly increased this cytokine compared to lysate alone. In addition, F1 plus lysate induced a greater release of TNF-α when compared to other experimental treatments (PnV, F2 and F3 plus lysate). On the other hand, F2 plus lysate significantly decreased the release of this cytokine compared to lysate alone and lysate plus PnV (Figure 6B). As for the release of IL-10, all treatments induced a significant increase compared to the untreated control. F1 and F3 plus lysate also significantly increased IL-10 compared to lysate alone and lysate plus PnV. Furthermore, F3 plus lysate induced a significant increase in this cytokine when compared to F1 plus lysate (Figure 6C).

Representative panels of morphological analyses are shown in Figure 6D–J. The control group (unstimulated DCs) showed round cell bodies, with no visible processes (panel D). After LPS treatment (positive control), cells with larger and irregular bodies, processes and granular cytoplasm were observed (panel E). Exposure to GL291 lysate induced enlarged cell bodies and the presence of processes (panel F). After treatment with lysate plus PnV or lysate plus fractions (F1, F2 and F3), DCs showed characteristics of mature cells, with more pronounced morphological changes when compared to lysate alone, such as larger cells, with irregular bodies and long processes (panels G–J). Interestingly, all experimental treatments (PnV, F1, F2 and F3) apparently induced less granular cells than LPS.

### 4.4. Evaluation and Selection of Molecules Purified from F1

**Figure 7:**
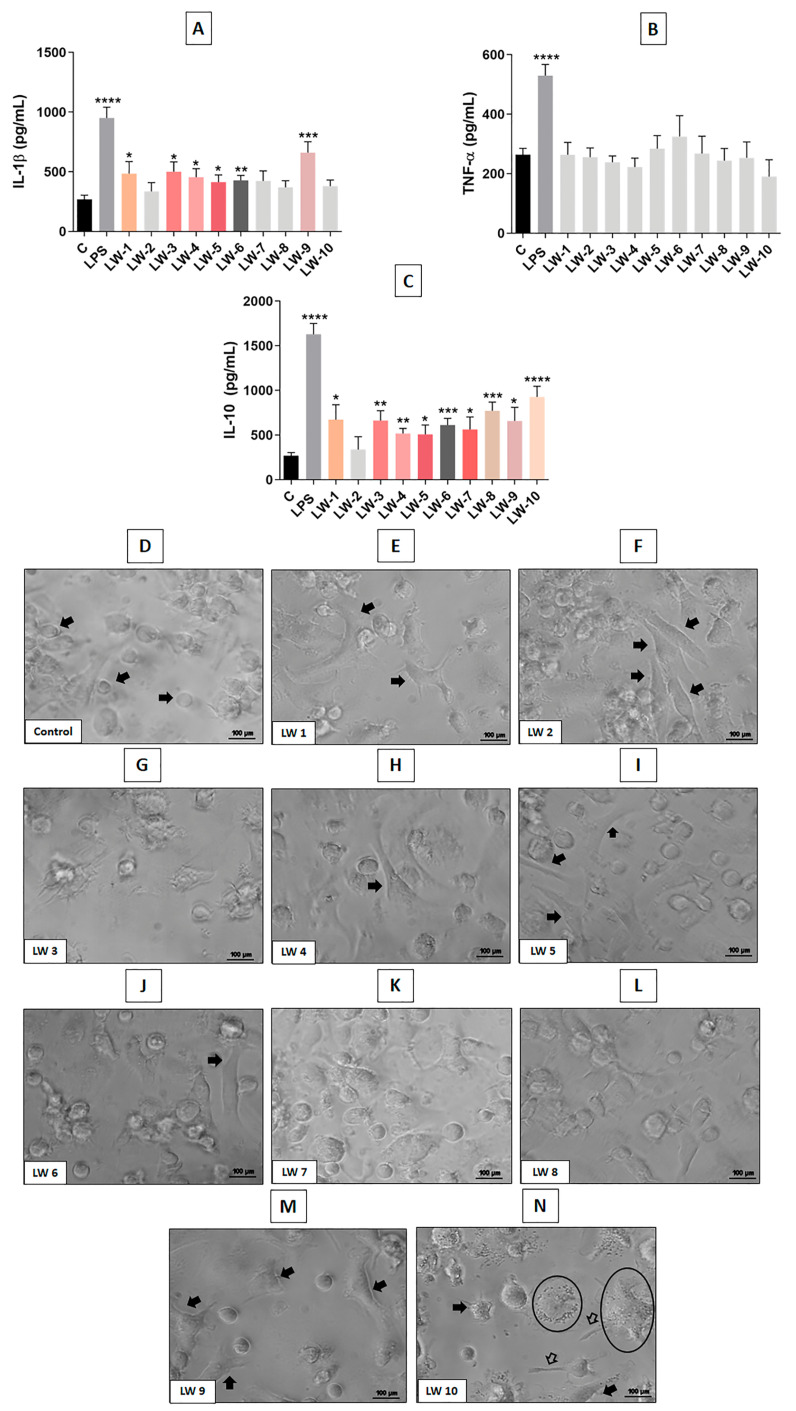
Cytokines release and morphology of DCs stimulated with purified subfractions. Graphs (**A**–**C**) show the production of pro-(IL-1β, TNF-α) and anti-inflammatory (IL-10) cytokines by DCs treated with purified subfractions (1 µg/mL) for 24 h, by ELISA. Panels D–N show morphological changes observed in DCs after treatments (**D**–**N**). Signs in panels indicate morphological changes compared to the untreated control (panel (**D**)). Arrows: irregular bodies and processes; circled cells: granular cytoplasm. Eclipse Ts2 Diascopic (Nikon Inc.); Bars = 100 µm. Data were analyzed by one-way ANOVA and Kruskal–Wallis test, presented as mean ± standard error (SEM). Unpaired Student’s *t*-test was used to compare each treatment with the control. * *p* < 0.05, ** *p* < 0.01, *** *p* < 0.001, **** *p* < 0.0001 compared to the untreated control. Results of three independent experiments.

Screening showed that F1 had more significant and balanced immunomodulatory effects on DCs when compared to the other fractions (F2 and F3), inducing a mature profile characterized by increased surface markers, enhanced TNF-α release and moderate IL-1β (which is a cytokine with pleiotropic effects on immune cells, angiogenesis, cancer cell proliferation, migration, and metastasis) and IL-10, in association with a classical activated morphological profile. Therefore, F1 was selected and subjected to further purification by HPLC, resulting in ten components referred as low weight subfractions (LW1–LW10) (Figure 7A).

Most LWs, but more pronouncedly LW9, were able to significantly increase IL-1β production by DCs compared to the untreated control. No LW significantly altered TNF-α release. On the other hand, several LWs, mainly LW6, LW8 and LW10, induced a significant increase in IL-10 release, compared to untreated cells (Figure 7A–C). While LW9 was the most efficient in increasing IL-1β, it induced moderate amounts of IL-10, showing a balance of these pro and anti-inflammatory cytokines.

Representative panels of morphological analyses are shown in Figure 7D–N. Unstimulated DCs (immature DCs) showed rounded cell bodies, with little or no process, as seen in panel A (control group). On the other hand, DCs exposed to treatment with LW1, LW2, LW4 and LW9 (panels E, F, H and M, respectively) presented enlarged and irregular bodies, with processes. Interestingly, DCs treated with LW10 showed marked morphological changes, with granular cytoplasm, in addition to irregular and enlarged bodies (panel N). However, the morphology of the DCs treated with LW3, LW5, LW6, LW7 and LW8 (most of them produced higher levels of IL10) was similar to that observed in the untreated control (panels G, I, J, K and L, respectively).

## 5. Discussion

In the present study, it was demonstrated that purified fractions from the venom of the *Phoneutria nigriventer* spider have immunomodulatory effects on DCs in vitro, which makes these molecules potential immunoadjuvants. The crude venom (PnV) was cytotoxic to DCs only at high concentrations and the fractions did not decrease cell viability at the concentration used. PnV had no significant effect on DC activation, while mainly F1 and F3 showed this action; this can be explained because the venom is a mixture of molecules that can have complex antagonistic effects. Therefore, when separated in fractions, F1 and F3 (low and high-weight molecules) were efficient in inducing a mature profile of DCs; both increased the release of IL-1β, TNF-α and IL-10, F1 augmented the expression of surface molecules involved in antigen presentation, and both fractions induced a typical activated morphology. These effects of F1 and F3 were much more prominent when the DCs were simultaneously exposed to tumor antigens (GB cell lysate).

Fractions plus lysate induced a more activated profile than when cells were exposed to PnV plus lysate, showing that, when separated, the components of the venom have amplified effects, confirming the hypothesis that antagonistic effects of molecules in the whole venom can occur. In addition, F1 plus lysate induced more TNF-α than lysate alone. These findings point to a potential role of venom components as adjuvants to a DC vaccine. The use of immunotherapy with a DC vaccine for solid tumors has not yet achieved satisfactory results [23]. Therefore, enhancing the antigen-presenting capacity of a DC vaccine, consequently improving clinical efficacy, has been the focus of several pieces of research.

In the present work, we chose to stimulate the DCs with tumor cell lysate. This is because it is known that loading DCs with tumor cell lysates positively regulates the expression of co-stimulatory surface molecules and increases the secretion of IL-6, IL-12 and TNF-α [24]. On the other hand, a vaccine containing a single antigen is limited and elicits MHCI responses but not MHCII and CD4+ T helper responses [25]. Meanwhile, tumor lysate or whole tumor cells contain a full complement of antigens, thus decreasing the risk of immune escape [25,26]. Compared to single tumor antigens, loading DCs with tumor lysates induces a stronger and more extensive immunological response against tumors [27,28,29]. In fact, the present data show that the GB cell lysates (human and murine) were very efficient in increasing DC activating markers, such as surface molecules expression and cytokines release.

These activation markers have been used to assess DC response. Studies have revealed the existence of distinct subsets of DCs that are highly specialized in boosting specific immune responses, and molecular and phenotypic characterization provides information about the DC signature. It is well established that mature DCs have a strong antigen presentation and immunostimulatory capacity by upregulating costimulatory molecules (such as CD40, CD80, CD83, CD86 and MHCII) [3,24,30]. Therefore, the present findings suggest that F1 induced a mature profile of DCs, as this fraction increased the surface molecules (mainly CD86) more than the GB cell lysate alone.

Furthermore, IL-2, IL-12, IFN-γ and TNF-α have been reported to stimulate CD4+ Th1 differentiation and accelerate Th1-mediated antitumor responses [31,32]; In addition, these cytokines stimulate CD8+ cytotoxic T lymphocytes (CTLs), the most important effector cells that recognize and eliminate tumor cells [33]. CTLs have long been associated with a good prognosis in many tumor types [34]. On the other hand, immunosuppressive tolerogenic DCs, which have down-regulated costimulatory molecules, are seen within the tumor mass and can suppress the induction of tumor-specific CTLs [33]. The present results, therefore, suggest that molecules present in F1, as this fraction increased TNF-α, induced a DC profile capable of differentiating Th1 and CTL, with potential antitumor application.

In addition, F1 induced a more balanced DC profile than F3, with more TNF-α and less IL-1β and IL10, and a moderately activated morphology; while F3 induced a highly activated profile, with large amounts of IL-1β and IL-10 and a very similar morphology to LPS-treated cells. Therefore, taken together, these results suggested that F1 contains molecules able to induce a balanced profile of activated DCs. For this reason, F1 was chosen to be purified again and their components were screened. LW9 induced a balance between pro- and anti-inflammatory cytokine release and an activated morphology of DCs. We recently demonstrated that the same molecule, LW9, induced an activated profile of macrophages [16]. It has been shown that PnV is composed mainly of small proteins, enzymes and peptides [35]. Herein, we confirmed the immunomodulatory potential of this molecule (LW9) and characterized it, showing that there is a peptide.

Over the past two decades, thousands of cancer patients have participated in clinical trials using therapeutic vaccines based on DCs loaded ex vivo with various formulations of tumor-associated antigens and adjuvants [23,36]. Dozens of phase I/II clinical studies with GB patients are ongoing or have been completed; however, no DC vaccine has been approved by the Food and Drug Administration (FDA) or other regulatory agencies around the world so far to treat GB. A DC vaccine, Sipuleucel-T (Provenge^®^), has been approved in 2010 for metastatic prostate cancer [36]. For GB, few trials have been published so far and the results have been encouraging but cannot provide robust evidence of clinical efficacy. Most assays used only DC pulsed with individual tumor lysate or with tumor peptides, DC loaded with autologous tumor homogenate, or DC cultured with individual tumor cells (for example, NCT04801147, NCT02049489, NCT04523688, respectively). The NCT00045968 trial [24] is a phase III study using a lysate-pulsed DC vaccine, demonstrating that the strategy has been safe and prolongs survival. The NCT01280552 trial [25] used DCs pulsed with six preselected synthetics derived from cancer stem cell associated antigen in GB; the therapy was well tolerated and increased progression-free survival by 2.2 months. Several factors, including the scarce number of DCs into and around the tumor and the immunosuppressive tumor microenvironment, contribute to the inefficacy of DCs as cell vaccines for GB [37,38].

Therefore, the effectiveness of these therapies might be improved by other strategies, such as combined therapies (for example, trial NCT04201873, which use the DC vaccine associated with the PD-1 antibody pembrolizumab) and the use of adjuvants that enhance the immunogenicity of DC vaccines [36,39]. However, there is no study, as far as we know, using adjuvants to improve DC activation/maturation in clinical trials. The NCT04201873 trial, currently under recruitment, will use i.m. administration of poly-ICLC, an immunostimulant (Toll-like receptor-3 agonist), concomitantly with the DC vaccine.

Thus, considering the effects of F1 on DCs presented in this study, including the augment in the CD86 costimulatory molecule, involved in antigen presentation, the increase of pro-inflammatory cytokines release (mainly TNF-α) and the induction of a typical activated morphology, it is suggested that molecules present in this fraction could be an opportunity to develop adjuvants to be used systemically in association with a DC vaccination or to enhance ex vivo DC maturation. The preliminary results presented suggest that LW9, a peptide, is a potential candidate for immunomodulation; however, further analyses are needed to confirm this effect. A study is underway to characterize the amino acid sequence and synthesize this component and perform preclinical tests.

## Data Availability

The datasets used and/or analyzed during the current study are available from the corresponding author upon reasonable request.

## Supplementary Materials

The following supporting information can be downloaded at: https://www.mdpi.com/article/10.3390/cells12071023/s1, Figure S1: Characterization of DCs generated in vitro; Figure S2: Chromatographic profile of isolated fractions from PnV by high-pressure liquid chromatography (HPLC).

## Abbreviations

ANOVA: One-way analysis of variance; APC: Antigen-presenting cell; CAR: Chimeric antigen receptor; CD: Cluster differentiation; CTL: Cytotoxic T lymphocyte; CTLA: Cytotoxic T lymphocyte-associated antigen; CEUA: Ethics Committee on the Use of Animals; DC: Dendritic cells; *E. coli*: *Escherichia coli*; ELISA: Enzyme linked immunosorbent assay; F: Fractions; FBS: Fetal bovine serum; FDA: Food and Drug Administration; GB: Glioblastoma; GM-CSF: Granulocyte-macrophage colony-stimulating factor; HPLC: High-pressure liquid chromatography; IL: Interleukin; IMDM: Iscove’s modified dulbecco’s medium; LPS: Lipopolysaccharide; MHC: Major histocompatibility complex; MTT: Thiazolyl blue tetrazolium bromide; NK: Natural killer; PBS: Phosphate buffered saline; PD: Programmed cell death; PDL: Programmed cell ligand; PnV: *Phoneutria nigriventer* spider venom; ROCK: Rho-associated protein kinase; RPM: Revolutions per minute; SEM: Standard error of the mean; TH: T-helper; TNF: Tumor necrosis factor.
