# Supplementary material for: Isolated Peptide from Spider Venom Modulates Dendritic Cells In Vitro: A Possible Application in Oncoimmunotherapy for Glioblastoma
Source: Cells. 2023 Mar 27;12(7):1023. doi: 10.3390/cells12071023 (PMC10092987; doi:10.3390/cells12071023)
Supplement: Supplementary file 1 [file cells-12-01023-s001.zip › cells-1897766-supplementary.pdf]

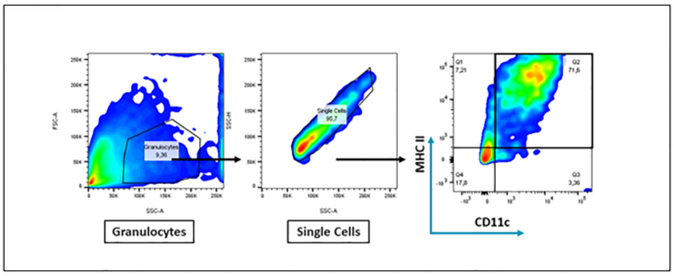
Granulocytes
Single Cells
MHC II
CD11c

Figure S1: Characterization of DCs generated *in vitro*. Dot Plot diagram representing the gate strategy for the analysis of double positive dendritic cells for classic myeloid DC surface markers (MHC-II and CD11c) by flow cytometry.

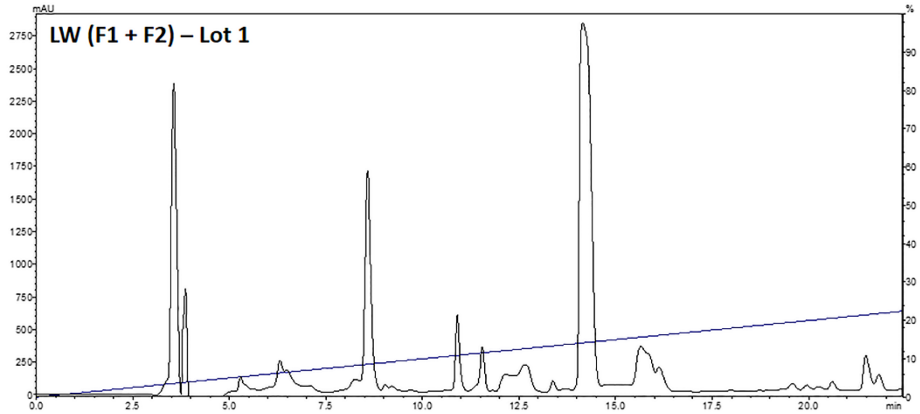
LW (F1 + F2) – Lot 1
mAU
%
min

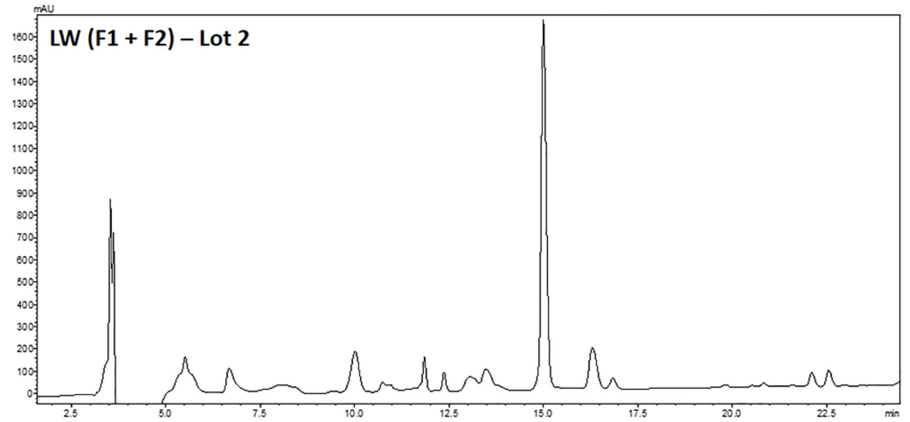
LW (F1 + F2) – Lot 2
mAU
min

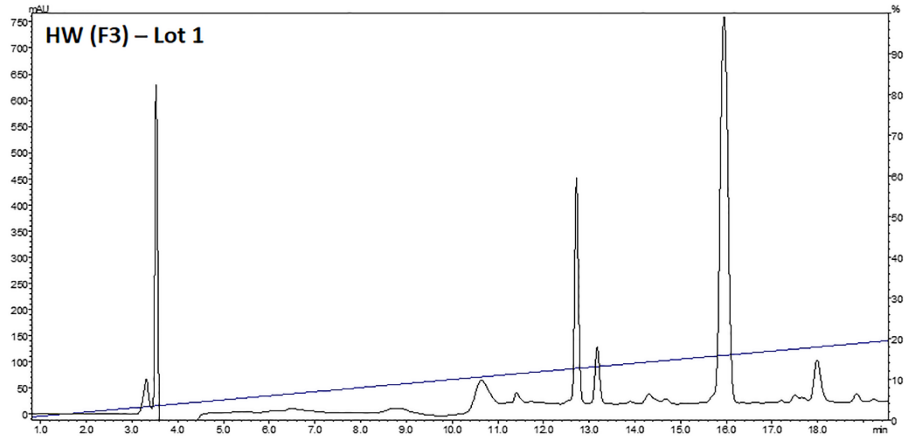
HW (F3) – Lot 1
mAU
%
min

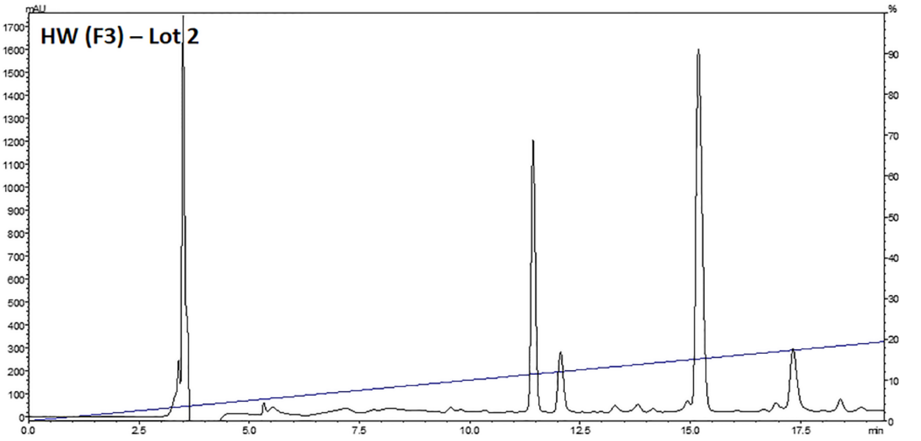
HW (F3) – Lot 2
mAU
%
min

Figure S2: Chromatographic profile of isolated fractions from PnV by high-pressure liquid chromatography (HPLC). Chromatographic profile of several pooled samples of fractions F1 and F2 (LW) and fraction F3 (HW), showing that the two batches of samples used in this work have similar compositions.
